# A sexual and reproductive health and rights journey: from Cairo to the present

**DOI:** 10.1080/26410397.2019.1676543

**Published:** 2019-11-08

**Authors:** Rebecca Brown, Eszter Kismödi, Rajat Khosla, S. Malla, Lucy Asuagbor, Ximena Andión-Ibanez, Sofia Gruskin

**Affiliations:** a Senior Director for Global Advocacy, Center for Reproductive Rights, New York, NY, USA.; b Chief Executive, Sexual and Reproductive Health Matters, London, UK; c Human Rights Technical Advisor, World Health Organization, Geneva, Switzerland; d Independent Consultant, Nepal; e Special Rapporteur on the Rights of Women in Africa, Yaoundé, Cameroon; f Program Officer, Ford Foundation Mexico, Mexico City, Mexico; g Professor of Preventive Medicine and Law, and Director, Program on Global Health and Human Rights, Institute for Global Health, University of Southern California, Los Angeles, California, USA

## Introduction

In the 25 years since the International Conference on Population and Development (ICPD), human rights legal standards have developed significantly, and those involved in sexual and reproductive health (SRH) programming have largely come to see the importance of rights to achieve their goals. Many national legal systems have made clear commitments and increased implementation in relevant areas, such as maternal health, abortion, sexuality education, sexual health, contraception, reproductive morbidities, gender-based violence, and with specific attention to the needs and rights of marginalised populations.

New challenges have nonetheless emerged, ranging from outright resistance and ideological attacks against gender equality and sexuality, to lack of political will and funding reductions. Rather than focus on these threats, this moment demands that we stand strong behind the human rights framework and legal guarantees that exist and ensure the positive role that rights play in programming and service delivery is championed.

## International and regional human rights standards

The strong human rights standards developed in the last 25 years have resulted in significant impacts on national law and policy-making, and programme implementation. These standards demand states take affirmative steps toward respecting, protecting, and fulfilling human rights including non-discrimination, health, life, freedom from ill treatment, gender-based violence and harmful practices, information, and privacy, as well as ensuring participation, transparency and accountability in how laws, policies and programmes are developed and implemented.^1^

Human rights standards have not only underscored the right to sexual and reproductive health as an integral part of the right to health, and in relation to the rights noted above, but also clarified their content and meaning. This includes that states must eliminate barriers to sexual and reproductive health services (e.g. parental or spousal/partner authorisation requirements), affirm the accessibility and affordability of services (e.g. contraception, including emergency contraception), and decriminalise sexual and reproductive health services (e.g. abortion), and states are to be held accountable if they fail. Importantly, with regard to adolescents, human rights bodies have noted a presumption of capacity to seek and have access to sexual and reproductive health services, including the ability to make autonomous and informed decisions regarding their reproductive health.^2^

Parallel to these international developments, strong human rights standards have emerged in many regions and countries. One landmark in this regard is the Maputo Protocol, which has had a significant impact on affirming sexual and reproductive health and rights across Africa. The Protocol has spurred health legislation and policies for improved access to services, increased health financing and investments, strengthened monitoring, evaluation and accountability, along with ensuring gender equality, women’s and girls’ empowerment, and respect of human rights.^3^ It also inspired further commitments and campaigns, such as the African Union’s Common Position on Ending Child Marriage, as well as the Saleema Initiative on ending female genital mutilation.

## Laws and policies

International human rights standards require states to bring their laws, regulations, policies and practices into line with established norms. The past 25 years have witnessed dramatic positive changes related to sexual and reproductive health. In part, this is due to increased evidence that harmonising laws with human rights standards can foster the promotion of sexual and reproductive health across and within populations, in contrast with the increasingly documented negative health impacts of laws that are in contradiction with human rights.^4^ For example, laws that foster the dissemination of comprehensive sexuality education contribute to people’s knowledge of what protects or damages their sexual and reproductive health, including where and how to seek further information, counselling and treatment if needed. On the other hand, laws that restrict access to health services by requiring third party authorisation for women and adolescents, or criminalise certain consensual sexual behaviours, restrict access and effectively exclude or deter people from seeking and receiving the information and services they require.

As a result, across the globe nearly 50 countries have liberalised their abortion laws; many have decriminalised same sex sexual conduct, most have adopted policies on free or low-cost maternal health care and contraception, and some have promulgated specific legislation on safe motherhood and sexual and reproductive health rights.^5,6^ The changes to national laws, for example on abortion, in Colombia, Ethiopia, Mexico City, Ireland and Chile resulted from long-standing efforts by national advocates on the ground and were supported by clear decisions and recommendations from UN and regional human rights bodies.^5^

National courts also play a significant role. In the Lakshmi Dhikta Case, for example, the Supreme Court of Nepal ruled women’s rights to health and life mean more than just keeping reproductive health services legal, but making them available to everyone.^7^ Following this case and others, Nepal adopted a national law on reproductive health in 2018 which operationalised reproductive rights standards through legislation.^8^ In Kenya, the Nairobi High Court’s most recent decisions affirmed that reducing maternal mortality and morbidity and ensuring access to free and respectful obstetric care for women are required to fulfil the rights to health and non-discrimination, and requires standards and guidelines for medical providers. It also affirmed the government obligation to take all necessary steps to ensure these rights through specific actions.^9^

## Implementation: sexual and reproductive health policy and programming

The ICPD has also had major impacts on international development policies. The Global Strategy on Women, Children and Adolescent Health and the Sustainable Development Goals, most notably Goal 5.6, are key examples of initiatives that incorporate human rights into sexual and reproductive health programming and implementation. At all levels, these initiatives have shed light on the processes and practices underpinning policy-making and programming.

Across the spectrum of sexual and reproductive health services, an emphasis can now be seen on the need for programmes to recognise the legal and policy environment where they are situated; not violate rights but consciously seek to contribute to their fulfilment; work towards the inclusion of those affected and most marginalised; and effectively operationalise the concepts of non-discrimination, participation and accountability. For instance, the government of Laos, along with partners, launched an initiative to provide client-friendly and free-of-charge family planning services to remote communities, working through community-based distribution agents. Results clearly show that when services are provided in this way, affected populations are more receptive to using them.^10^ Similarly, vital to improving monitoring of maternal deaths and promoting accountability, African Ministries of Health in Eastern and Southern Africa have worked towards institutionalisation of Maternal Death Reviews which have helped governments better plan interventions.

## Conclusion

Reflecting back, the legacy of Cairo can be seen in every region of the world. It is now well established that sexual and reproductive health policies, programmes and practices must be based on gender equality and human rights and ensure that everyone can exercise their rights related to sexual and reproductive health, free from discrimination, coercion and violence. However, the ICPD agenda remains *unfinished business,* with an increasing pressure simply to toe the line and reaffirm old commitments.

As most countries of the world move towards universal health coverage (UHC), making all efforts to ensure that sexual and reproductive health and rights are effectively incorporated becomes particularly important. As well as gender-based violence programming, humanitarian responses, and budgeting of sexual and reproductive health, the UHC package must include rights-affirming, comprehensive sexual and reproductive health services, including access to abortion, contraception, cervical cancer and sexuality related services, and ensure that the needs of populations whose behaviours or identities may be stigmatised or criminalised, are adequately addressed.

Since the ICPD, human rights have been applied in many different ways to improve sexual and reproductive health. A lot has been achieved but much remains to be done. Human rights are not a panacea but do play a crucial role in clarifying state obligations for the legal, policy and programmatic frameworks set out at the national level, interactions between governments and with civil society, and ultimately the actions states take within their own borders and beyond.

The review and appraisal of the Programme of Action is as much time for introspection as an opportunity for celebration. Human rights are essential for advancing the ICPD agenda, but ultimately crucial to ensuring the equality, autonomy, health, needs and aspirations of people around the world.
